# Single‐nucleotide polymorphism discovery and panel characterization in the African forest elephant

**DOI:** 10.1002/ece3.3854

**Published:** 2018-01-24

**Authors:** Stéphanie Bourgeois, Helen Senn, Jenny Kaden, John B. Taggart, Rob Ogden, Kathryn J. Jeffery, Nils Bunnefeld, Katharine Abernethy, Ross McEwing

**Affiliations:** ^1^ Agence Nationale des Parcs Nationaux Libreville Gabon; ^2^ WildGenes Laboratory The Royal Zoological Society of Scotland Edinburgh Zoo Edinburgh UK; ^3^ Biological and Environmental Sciences Faculty of Natural Sciences University of Stirling Stirling UK; ^4^ Aquaculture Pathfoot Building University of Stirling Stirling UK; ^5^ TRACE Wildlife Forensics Network Edinburgh UK; ^6^ Royal (Dick) School of Veterinary Studies & The Roslin Institute University of Edinburgh Edinburgh UK; ^7^ Institut de Recherche en Écologie Tropicale Libreville Gabon

**Keywords:** double‐digest restriction‐site‐associated DNA, forest elephant, Gabon, single‐nucleotide polymorphism

## Abstract

The continuing decline in forest elephant (*Loxodonta cyclotis*) numbers due to poaching and habitat reduction is driving the search for new tools to inform management and conservation. For dense rainforest species, basic ecological data on populations and threats can be challenging and expensive to collect, impeding conservation action in the field. As such, genetic monitoring is being increasingly implemented to complement or replace more burdensome field techniques. Single‐nucleotide polymorphisms (SNPs) are particularly cost‐effective and informative markers that can be used for a range of practical applications, including population census, assessment of human impact on social and genetic structure, and investigation of the illegal wildlife trade. SNP resources for elephants are scarce, but next‐generation sequencing provides the opportunity for rapid, inexpensive generation of SNP markers in nonmodel species. Here, we sourced forest elephant DNA from 23 samples collected from 10 locations within Gabon, Central Africa, and applied double‐digest restriction‐site‐associated DNA (ddRAD) sequencing to discover 31,851 tags containing SNPs that were reduced to a set of 1,365 high‐quality candidate SNP markers. A subset of 115 candidate SNPs was then selected for assay design and validation using 56 additional samples. Genotyping resulted in a high conversion rate (93%) and a low per allele error rate (0.07%). This study provides the first panel of 107 validated SNP markers for forest elephants. This resource presents great potential for new genetic tools to produce reliable data and underpin a step‐change in conservation policies for this elusive species.

## 1 INTRODUCTION

**Figure 1:**
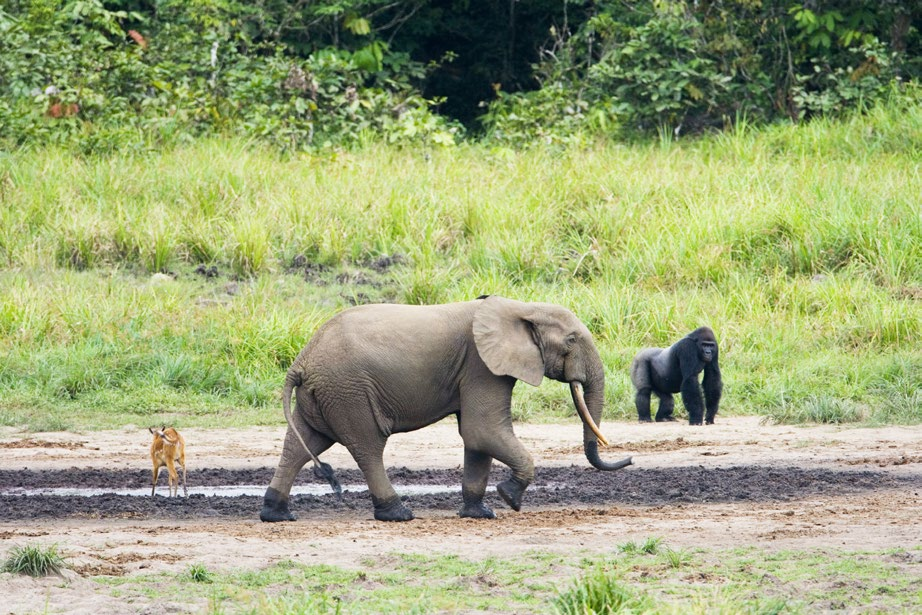
Forest elephant (*Loxodonta cyclotis*) at a forest clearing in Gabon (Photograph credit: David Greyo)

Evidences of lack of nuclear gene flow and high genetic divergence were used to split African elephants into two species, with the forest elephant (*Loxodonta cyclotis*) now established as a distinct species from the savannah elephant (*Loxodonta africana*) (Roca et al., 2015), even if not yet recognized as such by the IUCN African Elephant Specialist Group (AfESG). Due to its elusive nature and remote tropical rainforest habitat, compounded by a lack of species‐level recognition, the African forest elephant (Figure 1) has largely been understudied compared to the savannah elephant. Within the last decade, intense poaching and habitat reduction have caused a decline of more than 60% in Central African elephant numbers (Maisels et al., 2013). Gabon now hosts half of the remaining global population of *L. cyclotis*, but the northeast of the country suffered the steepest declines recorded for the decade 2004–2014 (Poulsen et al., 2017) and was revealed to be a major source of illegal ivory within Africa (Wasser et al., 2015). To respond to this conservation crisis, there is a desperate and immediate need to develop efficient tools to monitor forest elephant populations and threats.

Genetic tools have been widely used to understand elephant ecology and inform their management and conservation (Archie & Chiyo, 2012) and have shown tremendous potential to help understanding of the illegal ivory trade (Wasser et al., 2015). Numerous primers for presumed neutral genetic markers, including mitochondrial control region and microsatellites, are available in the literature for *L. africana* and the Asian elephant (*Elephas maximus*) (Ishida et al., 2012; Nyakaana, Okello, Muwanika, & Siegismund, 2005). However, nuclear genetic studies of *L. cyclotis* have all used microsatellite markers developed for *L. africana* (Eggert et al., 2014; Eggert, Eggert, & Woodruff, 2003; Johnson, 2008; Munshi‐South, 2011; Schuttler, Philbrick, Jeffery, & Eggert, 2014). While it is widely recognized that null alleles and size homoplasies may occur as a result of using microsatellite markers across species (Queloz, Duo, Sieber, & Grünig, 2010), only very recently were species‐specific microsatellite loci generated for *L. cyclotis* (Gugala, Ishida, Georgiadis, & Roca, 2016).

Microsatellites have long been the most widely used genetic markers in ecological studies, primarily due to their high mutation rate and polymorphism (Ellegren, 2004; Slatkin, 1995). However, technological advances are driving a shift in the field of molecular genetics from microsatellite to single‐nucleotide polymorphism (SNP) markers. Numerous studies have revealed the great potential for SNPs to be cost‐effective and highly informative markers (Helyar et al., 2011; Morin, Luikart, & Wayne, 2004; Vignal, Milan, SanCristobal, & Eggen, 2002), with a string of advantages including low error rates (Ranade et al., 2001), small amplicon sizes (<100 bp) (Senge, Madea, Junge, Rothschild, & Schneider, 2011), and technical portability and reproducibility across laboratories (Seeb et al., 2011). However, SNP resources for elephants are scarce, despite their high conservation profile and genome data being available for their development (Dastjerdi, Robert, & Watson, 2014; Elephant Genome Project 2017). To date, SNP markers have been used for species differentiation in African elephants (Ishida et al., 2011; Roca, Georgiadis, Pecon‐Slattery, & O'brien, 2001) and to study genetic diversity and structure of the highly endangered Bornean elephant (*E. maximus borneensis*) (Goossens et al., 2016; Sharma et al., 2012). However, novel genetic markers are urgently needed to better inform forest elephant conservation and management. The application of SNP markers to understand forest elephant population status and connectivity and the illegal ivory trade would tackle some priority areas of research.

The use of SNPs has been limited by the cost and availability of SNP discovery techniques, especially in nonmodel organisms. Recently, advances in next‐generation sequencing technologies and bioinformatics analyses have revolutionized the development of large numbers of genetic markers followed by the selection of a reduced high‐quality panel for a wide variety of species (Davey et al., 2011). Reduced representation genome sequencing approaches, where a subset of the genome is partitioned and sequenced, have arisen as inexpensive and simple methods for de novo SNP discovery in model and nonmodel species (Van Tassell et al., 2008). One of these approaches is the restriction‐site‐associated DNA (RAD) sequencing, which targets short fragments of DNA adjacent to a particular restriction enzyme site (Baird et al., 2008). The simplification of the procedure in the double‐digest RAD (ddRAD) approach, through the elimination of random shearing and the use of two‐enzyme digestion followed by strict size selection (Peterson, Weber, Kay, Fisher, & Hoekstra, 2012), has allowed discovery of targeted panels of a few thousand SNPs in a number of nonmodel species (e.g., Adenyo et al., 2017; Cruz et al., 2016). Notably, RAD methodologies permit simultaneous SNP discovery and genotyping. Where required, allele frequency data generated for multiple individuals from different locations can be exploited to better inform a subsequent targeted SNP assay design phase, reducing potential ascertainment bias (Clark, Hubisz, Bustamante, Williamson, & Nielsen, 2005; Nielsen, 2004).

In this study, we used ddRAD to discover thousands of potential SNP loci in the endangered forest elephant. Our aims were to (1) generate and identify potential SNP loci in forest elephants and (2) validate a subset of around a hundred SNP markers on a larger sample set via genotyping assays and comparison between genotyping and sequencing data.

## 2 MATERIALS AND METHODS

### 2.1 Samples

**Figure 2:**
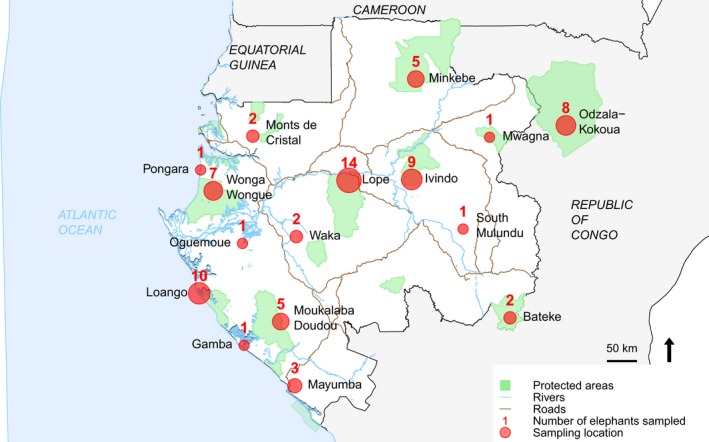
Distribution of elephant sampling localities throughout Gabon. The circles are proportional to the number of elephants sampled (with the total number indicated above). The number and location of samples used for the ddRAD analysis are given in Table 1

Sixty‐four samples from 58 forest elephants in Gabon were available for the SNP discovery phase. Blood, muscle, and skin samples were collected, as available, from 14 elephants immobilized for collaring operations in 2003 (Blake et al., 2008) and 44 elephant carcasses found in 14 locations (Figure 2). Samples were selected from a range of geographic locations across Gabon to reduce possible ascertainment bias (Nielsen, 2004). A second batch of 20 samples was added for candidate SNP validation. These samples were collected from six poached elephants in Gabon and eight elephants immobilized for collaring operations in the adjacent Odzala‐Kokoua National Park in Congo in 2014 (Figure 2). DNA was extracted primarily using the Qiagen DNeasy Blood and Tissue kit according to the manufacturer's protocol. In order to assess genotyping errors, 13 individuals were repeated using two different sample types and eight blood samples were extracted twice independently.

### 2.2 ddRADseq library preparation

DNA quality was assessed via agarose gel electrophoresis on a 1% gel, and only nondegraded DNA (as judged by a tight high‐molecular weight band against a lambda standard) was selected for the library preparation stage. DNA was quantified using a Qubit Broad Range dsDNA Assay (ThermoFisher Scientific) according to the manufacturer's instructions and normalized to c. 7 ng/μl.

A ddRAD library was constructed according to a modified protocol of the original Peterson et al. (2012) methodology. This is described in detail elsewhere (Brown et al., 2016; Manousaki et al., 2016). High‐quality DNA suitable for ddRAD library preparation was obtained for 23 elephants. An additional positive control (repeated individual, LOC0279_d) was included to allow for quality control of the experimental process and for assessment of genotyping error‐by‐read depth. Furthermore, each sample was processed in quadruplicate to enhance evenness of coverage of samples within the library. Briefly, individual genomic DNAs (24 × 4 replicates; 21 ng each) were restriction digested by *Sbf*I and *Sph*I, and then Illumina‐specific sequencing adaptors (P1 & P2) were ligated to fragment ends. The pooled samples were size selected (320–590 bp fragments) by gel electrophoresis, PCR amplified (15 cycles) and the resultant amplicons (ddRAD library) were purified and quantified. Combinatorial inline barcodes (five or seven bases long) included in the P1 and P2 adaptors allowed each sample replicate to be identified postsequencing. The ddRAD library was sequenced on the Illumina MiSeq Platform (a single paired‐end run; v2 chemistry, 2 × 160 bases).

### 2.3 Bioinformatics

The sequences were quality assessed using FastQC (Andrews, 2010), and the reads demultiplexed by barcode using the *process_radtags* module (default parameters) of the stacks bioinformatics pipeline (Catchen, Hohenlohe, Bassham, Amores, & Cresko, 2013). This module also filtered out low‐quality reads. The retained reads, now missing variable length barcodes, were then trimmed to a standard 148 bases in length. Demultiplexed read files were concatenated into read files for each individual (four barcode combinations per individual, see above). For each individual, matching forward and reverse reads were then concatenated into a single longer “artificial” read using a custom perl script. This was to allow for tracking of the closely linked read 1 and 2 loci in subsequent bioinformatics analyses.

The individual data were then processed using the *denovo_map.pl* module of stacks (m 10−M 2−n 1) to assemble and create a catalog of genetic loci contained in the data. The Stacks scripts *export_sql.pl* and *populations* and five filtering steps were used to retain all loci that fulfilled the following criteria:

1. Contained exactly one SNP (in the concatenated forward and reverse reads) to remove physically linked markers and ensure availability of a constant sequence surrounding the target SNP to facilitate primer design;
2. Contained exactly two alleles, as the presence of more than two alleles might represent repeat sequence found at multiple sites within the genome;
3. Were present in the data for ≥10 elephants and had a read depth of ≥10 reads per individual to maximize the likelihood of the SNP being real;
4. Were heterozygous in at least one individual but not in all individuals in the dataset; both the lack and apparent fixations of heterozygotes could be indicative of variation between repeat sequences found at more than one locus; and
5. Had a minimum of 50 bases flanking sequence either side of the SNP to ensure that the sequence meets the requirements for the design of a genotyping probe assay (LGC Genomics, 2014).

### 2.4 SNP validation

In order to validate the results from the bioinformatics pipeline, two sets of SNPs were tested for validation using different approaches. The default parameters were used for all programs, unless otherwise specified. First, a random subset of 22 SNP loci was selected as candidates for assay design and ordered from LGC Genomics using the Kompetitive allele‐specific PCR (KASP) system to evaluate the conversion rate that is the proportion of successful assays that resulted in distinct genotyping clusters. They were run on a StepOne real‐time PCR machine (Applied Biosystems) on the DNA samples used to generate the library. PCR was carried out in 8 μl single‐locus reactions following thermal cycling conditions recommended in the KASP user guide (LGC Genomics, 2013). The quality of the genotyping cluster plots was visually assessed. When the probe did not produce distinct clusters, further examination of the SNP containing sequences was conducted by aligning them against the *L. africana* genome (LoxAfr 3.0, Genbank Assembly ID: GCA_000001905.1, July 2009, Elephant Genome Project) using NCBI's Basic Local Alignment Search Tool (BLAST) to investigate any repetition within the genome.

Second, a genotyping panel was selected among the candidate SNP markers using a combination of measures of genetic diversity and divergence, in order to validate assay performance and select potentially informative markers with the aim to explore genetic variation among individuals and populations. The filtered matrix of sequencing genotype data at 1,365 loci was examined for “missingness” using PLINK (Purcell et al., 2007). A principal components analysis was run using the package *adegenet* (Jombart, 2008) in R (R Core Team 2016) to examine structure in the data matrix (results not shown). Three population clusters were then defined based on a mixture of the geographic and genetic information: North‐East (South Mulundu, Ivindo, Minkebe, Monts de Cristal), Central (Lope, Waka), and Coastal (Wonga Wongue, Mayumba, Loango, Moukalaba Doudou) (Figure 2). These groups were used to calculate and rank loci according to expected heterozygosity (*H*_E_), global *F*_ST_, and *F*_ST_ in the three pairwise population combinations. Loci were then given an unweighted joint rank across all five categories, and the highest ranking 266 SNPs were chosen. Finally, loci were excluded that had zero or >1 BLAST matches against the *L. africana* genome using a discontiguous megablast of the 148 bases sequence containing the SNP. The cutoff *e*‐value was set at 10^−10^ with a minimum alignment length of 100 bp including the SNP site. Sequences with no matches based on these criteria were excluded on the basis that they could be from a different organism, while multiple matches revealed that the sequence was duplicated within the genome and therefore not suitable for assay design. The 30‐bp flanking sequences either side of the SNP were also independently searched against the savannah elephant genomic data (cutoff *e*‐value <0.00001 and length >27 bp) to minimize the chance of designing primers that may anneal at multiple sites. This step was added following validation of 22 probes from the pipeline (see above).

Sequence information for 115 SNP loci that passed the above criteria was submitted to LGC Genomics service laboratories for KASP assay design and genotyping of 74 forest elephant DNA samples that included both the samples used to generate the library and all additional samples that yielded suitable DNA (as revealed by DNA quality and quantity tests) even if they were not suitable for the ddRAD library construction. The stringent parameters used by LGC Genomics for automatic allele calling usually result in a high proportion of unassigned genotype calls (Semagn, Babu, Hearne, & Olsen, 2014). Therefore, the genotype plots of each assay were visually checked using SNPviewer 2 software (LGC Genomics) and rescored manually if individuals that clearly belonged to a cluster had not been called automatically. The proportions of manually rescored genotypes and missing data (no calls) were calculated for each locus as indices of assay quality. Genotype profiles obtained from the KASP assays were compared to the genotype data from the ddRAD pipeline to ensure that matching genotypes were recovered. We distinguished two types of mismatches: (1) category 1—a SNP scored as heterozygote by KASP genotyping assay but homozygote by sequencing; and (2) category 2—a SNP scored as homozygote by KASP genotyping assay but heterozygote or a different homozygote by sequencing. A proportion of category 1 mismatches were to be expected because allelic dropout usually occurs during RAD sequencing (Gautier et al., 2013) and increases for low read coverage loci (Pelak et al., 2010). Category 2 mismatches were likely due to sequencing artifacts or assay design failure, and these SNP loci were removed from consideration. For all converted assays, the allelic error rate, including false alleles and allelic dropout, was estimated from mismatches between the genotypes of repeated individuals. Two positive controls were genotyped seven times. In addition, 12 individuals were repeated twice using DNA extractions from both tissue and blood or saliva samples, and DNA was extracted twice independently from eight blood samples. Preliminary measures of polymorphism and population differentiation were estimated using the dataset of 57 individuals attributed to one of the three predefined populations (North‐East, Central, and Coastal). Minor allele frequency (MAF) and expected (*H*_E_) and observed heterozygosity (*H*_O_) were estimated for each population using the R package adegenet (Jombart, 2008), and overall *F*_ST_ was calculated in the R package *pegas* (Paradis, 2010).

### 2.5 Characterization of the loci

In the absence of a reference genome for forest elephants, the selected loci were searched against the African savannah elephant *L. africana* assembly. A megablast of the 148 bp sequences containing the SNP (*e*‐value cutoff = 10^−40^) was used to match the sequences to scaffolds and determine if the SNPs were located within a gene locus, and in particular within a coding region. Pairwise linkage disequilibrium was tested for using the R package *LDheatmap* (default parameters) (Shin, Blay, McNeney, & Graham, 2006).

## 3 RESULTS

Approximately one‐third of the samples yielded DNA of sufficiently high‐molecular weight to attempt ddRAD library preparation. In total, 17,378,607 raw sequencing reads were generated from the 24 sample library, representing individuals from 10 locations (Table 1). Three individuals (LOC0044_a, LOC0225_a and LOC0394_a) had very low read numbers (<12,000) and were removed from further bioinformatic analyses at this point. Another individual (LOC201_a) was excluded because, despite exhibiting the highest read depth, it had missing data at all loci, which was likely due to pre‐DNA extraction contamination of the sample (bacterial decay). The average read depth per individual for the remaining samples was 656,955 (range: 112,534–1,259,614). The data for each individual are deposited in the NCBI Short Read Archive under accession numbers SRR6371502‐21. A catalog of 31,851 tags was assembled, of which 4,749 contained exactly 1 SNP with exactly two alleles and 1,365 met the chosen population coverage and read depth requirements (Appendix S1). A further 161 of these SNPs were removed from consideration because of a lack of heterozygotes, and 784 were not suitable for assay design (the SNP was less than 50 bp from either end of the read). This resulted in a dataset of 420 SNP loci for 19 elephants.

**Table 1:** Sampling locality and number of ddRAD reads generated per individual, following quality filtering and concatenation

Sample ID	Population	Number of reads
LOC0279_b	South Mulundu	659,295
LOC0279_d (positive control)	South Mulundu	788,139
LOC0049_a	Ivindo	735,621
LOC0050_b	Ivindo	908,474
LOC0051_a	Ivindo	566,824
LOC0225_a	Loango	11,450
LOC0274_a	Loango	791,494
LOC0037_a	Lope	1,159,937
LOC0038_a	Lope	1,088,247
LOC0088_a	Lope	633,191
LOC0044_a	Mayumba	128
LOC0201_a	Mayumba	2,264,818
LOC0309_a	Mayumba	501,070
LOC0035_a	Minkebe	453,030
LOC0121_a	Minkebe	112,534
LOC0122_a	Minkebe	566,704
LOC0311_a	Monts de Cristal	595,430
LOC0127_a	Moukalaba Doudou	120,598
LOC0151_a	Moukalaba Doudou	1,002,779
LOC0310_a	Moukalaba Doudou	133,832
LOC0041_a	Waka	683,264
LOC0263_a	Wonga Wongue	1,259,614
LOC0394_a	Wonga Wongue	1,095
LOC0040_a	Wonga Wongue	379,030

All samples used for discovery were tissue (skin and muscle) samples, except LOCO279_d which is a duplicate blood sample used as a positive control in the library.

**Figure 3:**
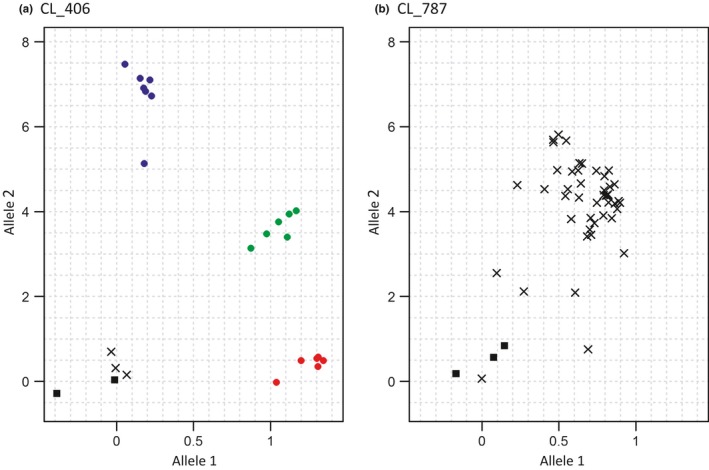
Examples of genotype plots using validated and failed KASP assays. The fluorescence for the two alleles is plotted along the *x*‐ and *y*‐axes. (a) Samples were well separated into three clusters using assay CL_406, with the green, blue, and red dots representing the heterozygous and the two homozygous genotypes, respectively; black squares are negative controls; and crosses are ungenotyped samples. (b) The second assay CL_787 produced a single diffuse cluster and failed to define genotypes. BLAST searches against *Loxodonta africana* genome produced a unique match for CL_406 and multiple matches for CL_787

A moderate conversion rate of 68% was achieved with the first set of 22 randomly chosen SNP loci. Fifteen KASP assays yielded scorable profiles, whereas seven produced diffuse clusters that could not be confidently resolved into genotypes (Figure 3). BLAST alignment against the *L. africana* genome revealed that this could generally be explained by the likely presence of potential multiple primer binding sites in the genome.

A further three individuals (LOC0121_a, LOC0127_a and LOC0310_a) were removed from the dataset at this stage due to having high levels of missing data in the matrix (>70%), leaving a dataset of 420 SNPs and 16 individuals with >60% of the loci genotyped. A list of 266 highest ranking SNPs was then selected according to the measures of genetic diversity and divergence (see above). A BLAST search of the whole sequence and of the flanking regions of the SNP against the *L. africana* genomic data produced no matches for 36 of these loci and multiple matches for 39 others. The search identified a unique match based on selected criteria for 191 loci, of which a random subset of 115 SNPs was subsequently chosen for KASP assay design and genotyping.

Following genotyping of 74 samples, six SNPs (CL_2059, CL_2174, CL_3260, CL_5749, CL_6220, CL_10063) failed to provide distinct clusters in the signal intensity plot and were excluded from further analysis. When comparing the genotypes obtained from the KASP assays to the 19 ddRAD profiles, the proportion of missing data was higher in the ddRAD pipeline (23.0%) than in the LGC genotyping data (1.7%). The proportion of category 1 and category 2 mismatches was 1.40% and 0.15%, respectively. Only three loci yielded category 2 mismatches, of which one (CL_340) was rescored as the discrepancies were due to KASP scoring error caused by low‐quality plots, namely little separation between the heterozygous group and one of the homozygous groups. The two other loci (CL_3004 and CL_10172) were removed from consideration because of a high proportion of category 2 errors (9.26% and 6.82%, respectively). This resulted in an estimated conversion rate of 93% (107 of 115).

In total, 2.6% of the genotypes were manually rescored. The allelic error rate among replicates was 0.07%. The overall quality of the genotyping plots was good (i.e., clearly segregated clusters), as even though 73% of SNPs (78 of 107) needed to be rescored for at least one sample, only 16 were rescored for more than 5% of the samples (range: 0%–17.2%). The proportion of missing genotype data per locus was <15% for all except 13 loci (overall range: 2.2–44.1) (Table S1). Mean MAF for individual loci was 0.213, and 30.3% of SNPs were highly polymorphic (MAF > 0.3). Fifteen loci were monomorphic in at least one of the three populations. Mean overall *H*_O_ and *H*_E_ per locus were 0.27 and 0.31, respectively. Mean overall *F*_ST_ was 0.015, suggesting low genetic differentiation, but ranged from 0.03 to 0.162 for 31 SNPs, indicating substantial differences in allele frequencies at these loci (Table S2). However, these measures are preliminary due to the small sample size.

### 3.1 SNP characterization

**Figure 4:**
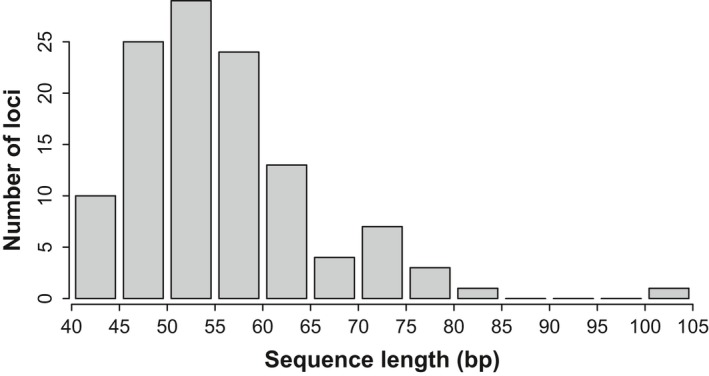
Distribution of sequence length following assay design for the 107 validated SNPs. The median length was 54 bp and ranged from 41 to 104 bp. Only two assays targeted a sequence of more than 80 bp

Following assay design, the median length of the targeted sequence, as obtained from matching forward and reverse primers to the 148 bp sequences containing the SNPs, was 54 (range: 41–104) (Figure 4 and Table S1). All 107 SNP sequences were successfully mapped to one of 60 *L. africana* unplaced scaffolds (sequence similarity from 97% to 100%), of which 78 SNPs (71.6%) matched the same scaffold as one to five other SNPs suggesting that they could be linked (Table S3). However, linkage disequilibrium was not detected between most loci. Only four pairs were in weak linkage disequilibrium (*r* ^2^ > .3), but the two loci in each pair did not belong to the same scaffolds. In total, 50 sequences (46.7%) returned a match against a functional region of the *L. africana* genome, of which only seven SNPs occurred within the coding DNA sequence of the gene (Table S3).

## 4 DISCUSSION

After quality filtering, we have generated a new genetic resource of 1,365 SNP loci which is available for further studies. As this is the first genome‐wide set of SNP markers generated for African elephants, it represents a major advance for the genetic study of this taxon.

In this study, ddRAD was demonstrated to be effective for the rapid discovery of a large number of SNPs in the forest elephant. Due to double restriction digestion and precise size selection, ddRAD sequencing produces only the subset of fragments generated by cuts with both restriction enzymes and close to the target size. Therefore, ddRAD libraries are expected to provide less coverage than the original RAD method (Peterson et al., 2012). In addition, we used concatenated tags during the filtering process in order to preserve linkage information from both reads and create a high‐quality dataset. This approach reduced the final number of SNPs generated compared to studies handling forward and reverse sequences separately and was compounded by the strict first filtering criterion to allow just a SNP per tag. As a result, the first two filtering steps led to a sharp reduction of 85.1% in the number of loci retained. As a comparison, ddRAD sequencing and SNP filtering using restrictive criteria similar to ours generated 3,060 SNPs in koala (Kjeldsen et al., 2016) and 2,381 in an Oriental fruit bat (Chattopadhyay et al., 2016). Differences are likely linked to lower number of individuals and read depth in the forest elephant discovery panel. Both the abovementioned studies used a large sample size (46 and 171, respectively) and reported an average of approximately 1.8 million reads per individual, which is three times higher than in our study.

A major limitation for the preparation and success of this library was the difficulty in obtaining high‐quality DNA samples from an endangered and elusive species. Whereas other studies used fresh blood and tissue samples, we used tissue samples obtained from carcasses of elephants poached for ivory, killed accidentally, or shot during crop raiding to generate the library. Tropical environments often lead to high degradation rates of genetic material in carcasses. Thus, even though 64 samples were available at the stage of the library preparation, 41 were removed from consideration due to poor DNA quality. In order to obtain a good‐quality set of SNP markers, a major component of the SNP discovery phase is to choose a panel of samples of diverse origin to minimize any ascertainment bias (Clark et al., 2005). The use of a narrow sample size from selected populations for a discovery process may result in a bias toward highly polymorphic SNPs or SNPs that segregate within particular populations, especially if population structure is pronounced (Clark et al., 2005). Our final selection of 23 samples was therefore a compromise between DNA quality and sample location across the country in order to avoid as much as possible any ascertainment bias toward particular populations while retaining overall sample size. However, a further four individuals were removed from consideration due to DNA degradation, as suggested by a high rate of missing data from ddRAD.

A high proportion (~70%) of the loci containing exactly one SNP were removed from consideration because of the generally low read depth per individual at a locus, leading to a high rate of missing data among individuals. In retrospect, as the elephant genome is large, with a size between 3.1 and 4.01 Gb (LoxAfr 3.0, Elephant Genome Project; Kasai, O'Brien, & Ferguson‐Smith, 2013), a narrower size selection or more sequencing effort might have produced better read depth per locus and resulted in more loci kept in the filtering stages. Strict filtering criteria decrease the genotyping error rate but also tend to reduce the amount of data retained. Previous studies recommended the use of a sequence read depth of between 30–35× for accurate genotyping due to the high risk of sequencing errors, mainly allelic dropout, when the read depth decreases (Pelak et al., 2010). Fountain, Pauli, Reid, Palsbøll, and Peery (2016) reported that, in a de novo‐assembled dataset, increasing the coverage threshold from 5× to 30× decreased the frequency of genotyping errors from 0.11 to 0.04, but also led to a 13‐fold decline in the number of loci detected across individuals. The coverage threshold should be a balance between acceptable risk of errors and amount of data generated, in light of the objectives of the study. Our study used sequencing data to discover potential SNPs, but not for estimating some population genetic parameters, except for the purpose of selecting a reduced SNP panel. Therefore, the major challenge was not to reduce the amount of allelic dropout within the data but to avoid selecting false SNPs. The chosen threshold of 10× coverage appeared to be a sensible balance that retained about 30% of the potential SNPs while generating a low allelic error rate (1.52%). It was combined with a subsequent laboratory validation of a subset of SNPs to confirm them being real.

We validated genotyping assays for a subset of 107 SNP loci. KASP assays have been successfully used in a variety of crop and animal species (e.g., Hiremath et al., 2012; Senn et al., 2013), and they generally demonstrate high conversion rates and low error rates among replicates. The allelic error rate among replicates for the elephant SNPs was particularly low (0.07%), in contrast to the 0.7%–1.6% reported for other studies using this technology (Semagn et al., 2014). Conversion rate was high, with the additional BLAST alignment check against *L. africana* genomic data improving the conversion rate from 68% to 93%. This illustrates the value of whole‐genome data for assisting with such studies and pointed to variation between sequence repeats found at multiple sites within the genome being probably the main factor explaining SNP conversion failure. Two SNP assays (CL_3004 and CL_10172) were removed from consideration because they did not cluster as expected genotypes. Monomorphic results were observed in the cluster plots, whereas all three genotypes existed in the ddRAD data. This was likely due to sequence repeats that were not detected using the incomplete *L. africana* genomic data. Even though ddRAD sequencing is suitable for nonmodel organisms, these results highlighted the advantages of using genetic resources from a closely related species to detect sequence repeats. *L. africana* genomic data have also successfully been used to characterize SNP markers in the Bornean elephant (*E. maximus borneensis*) (Sharma et al., 2012) and microsatellites in the forest elephant (Gugala et al., 2016). If no related genome is available, the number of loci selected for assay design should be increased in order to take account of expected lower conversion rate.

One major challenge was to find SNPs that were appropriate for assay design, as our criterion (50‐bp flanking region upstream and downstream of the target SNP) removed almost 58% of loci from consideration. A similar issue has been raised by another study that reported that as many as 75% of potential SNPs were unsuitable for assay design (Sharma et al., 2012). We followed LGC Genomics recommendations for KASP assay design, but these criteria are stricter than other genotyping platforms. A minimum of 50 bases of sequence on either side of the target SNP is required for submission of KASP assay design, similar to Illumina GoldenGate, compared with 40 bases for Applied Biosystems TaqMan assays and down to 30 bases with Sequenom iPlex assays for instance. Following assay design, the median length of the targeted sequence was as small as 54, meaning that if it was possible to relax this filtering parameter, more potentially assayable SNPs could be retained. Alternatively, using longer sequencing read technology, for example, 250 bases paired‐end sequencing, would generate more SNPs with 50 bases flanking regions around the SNP position.

From a practical perspective, potential useful applications for this new set of 1,365 markers include individual identification, parentage analysis, population genetics analysis, and identification of the source of seized ivory. Genetic tools are particularly attractive for individual‐level studies in elusive forest species. In addition, a thorough understanding of population genetic structuring of forest elephants is essential to effectively manage populations across the species range. Given the limited sample size, using *F*_ST_ on populations of five to six individuals potentially introduced bias in SNP panel selection. However, this method was used to identify markers that might be showing population differentiation. The 107 validated SNPs will be re‐assessed for utility in future population structure analysis, which may require the validation of additional loci to reach enough power. Particular attention will be paid to several of the newly developed SNP markers that were located within the coding region of genes, as markers associated with gene under selection may increase the power to detect population differentiation (Landguth & Balkenhol, 2012). Preliminary analyses of MAF and heterozygosity (Table S2) indicated that many of the 107 SNP markers will be useful for individual identification and parentage analysis within Gabon. However, further investigation is needed to explore the extent of genetic variability at these new SNP markers in other forest elephant populations. Ascertainment bias is a major challenge in the widespread use of SNP panels, even though corrections have been proposed (Albrechtsen, Nielsen, & Nielsen, 2010). The samples used in this study were widely distributed throughout Gabon, but the SNP markers developed in Gabon are expected to underestimate genetic diversity in other range countries, so they should be applied to the examination of population structure with care. However, the genetic structure of forest elephant populations in Central Africa is expected to be weak (Johnson, 2008) due to relatively high mobility of individuals, suggesting that with some further testing on populations outside of Gabon, these markers may have wider use for individual ID across the species range. In contrast, preliminary testing of our 107 SNPs in two African savannah elephant samples and BLAST alignment of these alleles to the published *L. africana* assembly found only two markers to be polymorphic (data not shown), which is consistent with the species separation (Ishida et al., 2011).

## 5 CONCLUSION

We generated the first genome‐wide SNP resources for forest elephants that are available for further studies. In addition, we validated KASP assays for a subset of 107 SNPs to allow in‐house genotyping in local laboratories that have limited access to sequencing technologies. The use of this novel SNP panel on a wider range of samples will provide the foundation for new practical tools and in‐depth information for the conservation and management of forest elephants. Given the urgency of conservation and management interventions for this species, we believe that research on the population status, genetic structure, and the illegal ivory trade of forest elephants would greatly benefit from a shift toward use of SNP markers to increase potential for data sharing between researchers and allow the rapid expansion of databases in time and space required for timely response to the current crisis in this species’ survival prospects.

## CONFLICT OF INTEREST

None declared.

## AUTHOR CONTRIBUTIONS

S.B., H.S., R.O., and R.M. designed the study. S.B. collected the samples. S.B. and J.K. performed laboratory work. J.B.T. designed ddRAD protocol and performed the sequencing run. R.M. supervised laboratory work. H.S. performed bioinformatic analyses. S.B. and H.S. performed statistical analyses. S.B. and H.S. wrote the manuscript. H.S., J.K., R.O., K.J., N.B., K.A., J.B.T, and R.M. revised the manuscript. H.S., K.J., N.B., K.A., and R.M. supervised the study.

## DATA ACCESSIBILITY

The data for each individual are deposited in the NCBI Short Read Archive under accession numbers SRR6371502‐21 (study SRP126637). Details for all SNPs and validated primers are found in Appendix S1 and Table S1.
